# Genitourinary tuberculosis

**DOI:** 10.4103/0970-1591.42617

**Published:** 2008

**Authors:** Narmada P. Gupta

**Affiliations:** Department of Urology, All India Institute of Medical Sciences, New Delhi – 110 029, India. E-mail: narmadagupta@gmail.com

Tuberculosis is the cause of unaccountable human sufferings and economic loss and is one of the earliest human afflictions. Genitourinary tuberculosis (GUTB) term was coined by wildbolz in 1937 and comprises 20% of all extra pulmonary tuberculosis. It was considered that we have conquered this disease, but due to HIV infections and drug resistance, there is a resurgence of tuberculosis. Though the incidence has decreased in the developed countries, it is still highly prevalent in the developing and underdeveloped nations.

In text books of urology written in the USA and Europe, the description of this disease is shrinking. The Editorial based on IJU is to be complimented for publishing the supplement on GUTB. I am grateful to them for entrusting me to edit this supplement. The topics were carefully selected to cover this disease in all perspectives. I am grateful to all my authors who worked hard and had written the chapters after reviewing the existing published literature. I am hopeful that this supplement of the Indian Journal of Urology will fill the void in this subject and will be useful for the postgraduates and urologists for better management of these unfortunate patients afflicted by GUTB.

